# Structure and Electromagnetic Properties of Cellular Glassy Carbon Monoliths with Controlled Cell Size

**DOI:** 10.3390/ma11050709

**Published:** 2018-05-01

**Authors:** Andrzej Szczurek, Vanessa Fierro, Artyom Plyushch, Jan Macutkevic, Polina Kuzhir, Alain Celzard

**Affiliations:** 1 Université de Lorraine, CNRS, IJL, F-88000 Epinal, France; jahman19@wp.eu (A.S.); vanessa.fierro@univ-lorraine.fr (V.F.); 2 Center of New Technologies, University of Warsaw, Banacha 2c, 02097 Warsaw, Poland; 3 Research Institute for Nuclear Problems of Belarusian State University, Bobruiskaya Street 11, 220030 Minsk, Belarus; artyom.plyushch@gmail.com (A.P.); polina.kuzhir@gmail.com (P.K.); 4 Faculty of Physics, Vilnius University, Sauletekio 9, LT-10222 Vilnius, Lithuania; jan.macutkevic@gmail.com; 5 Department of Radiophysics, Tomsk State University, 36 Lenin Prospekt, 634050 Tomsk, Russia

**Keywords:** cellular monoliths, glasslike carbon, porosity, electrical conductivity, permittivity, electromagnetic properties

## Abstract

Electromagnetic shielding is a topic of high importance for which lightweight materials are highly sought. Porous carbon materials can meet this goal, but their structure needs to be controlled as much as possible. In this work, cellular carbon monoliths of well-defined porosity and cell size were prepared by a template method, using sacrificial paraffin spheres as the porogen and resorcinol-formaldehyde (RF) resin as the carbon precursor. Physicochemical studies were carried out for investigating the conversion of RF resin into carbon, and the final cellular monoliths were investigated in terms of elemental composition, total porosity, surface area, micropore volumes, and micro/macropore size distributions. Electrical and electromagnetic (EM) properties were investigated in the static regime and in the Ka-band, respectively. Due to the phenolic nature of the resin, the resultant carbon was glasslike, and the special preparation protocol that was used led to cellular materials whose cell size increased with density. The materials were shown to be relevant for EM shielding, and the relationships between those properties and the density/cell size of those cellular monoliths were elucidated.

## 1. Introduction

Cellular carbons, and especially carbon foams, are considered for a large number of applications due to their unique properties [1]. Their attractiveness lies in remarkable features such as low density and correspondingly huge porosity, which is associated with interesting thermal and mechanical properties. Carbon foams, whatever their nature (reticulated or cellular), are therefore of interest from many fundamental, industrial, and technological points of view. For instance, reticulated carbon foams with fully open, polyhedral cells from which only struts remain are known as thermal [2,3] and acoustic [4] insulators, and function as supports for photocatalysis [5] or as materials for electrodes [6,7]. Cellular carbon foams present higher mechanical properties than reticulated carbon foams of similar density, since the porous structure of the former is far less open than that of the latter, and given that their cells are connected with each other through much smaller windows. Cellular carbon foams have been suggested as impact absorbers [8,9], as materials for electromagnetic applications [10], and also as thermal insulators [4,11,12].

Although useful properties of carbon foams such as electrical conductivity and mechanical resistance are developed during the pyrolysis of their organic precursors, key characteristics such as very low density and high porosity are controlled by synthesis conditions. For instance, the structural properties of phenolic rigid foams, which are precursors of glasslike carbon foams, are controlled by the formulation, e.g., through the amount of blowing agent [13,14] or the initial concentration of resin [12], as well as by the foaming conditions. Usually, a clear correlation between synthesis conditions and final properties is observed.

Another now well-known method for preparing cellular materials is based on the use of templates for generating the porosity. Templates may be “hard” or “soft”. Hard templating is aimed at building a porous structure based on various kinds of supports such as polymeric skeleton, e.g., polyurethane foams [15] or household cleaning pads [16], or particles of controlled, known sizes, such as poly(methyl methacrylate) (PMMA) particles [17], and recovering a carbon replica by pyrolysis. On the other hand, soft templating, which is based for example on emulsions such as polymerised high internal phase emulsions (polyHIPEs), leads to a porous structure controlled both by the concentration of compounds in the formulation and preparation conditions [18,19,20,21]. The resultant highly porous carbon monoliths generally present lower average cell sizes than for typical carbon foams.

In the present work, hard templating was used to prepare cellular glassy carbons that had as much of a controlled cell size as possible. For that purpose, resorcinol formaldehyde (RF) xerogel and paraffin beads were used as carbon precursor and as templates, respectively. Paraffin beads were chosen because they are easy to produce with a size that can be controlled by tuning the stirring speed during preparation, which is followed by sieving. After use as templates in the RF resin, these beads can be simply removed by washing in petroleum ether or by melting in a hot water bath. Additionally, it is known that when using RF resin as carbon precursor, various micro and/or mesoporous structures can be obtained by adjusting the synthesis conditions [22,23,24]. As paraffin beads are responsible for the macroporous structure of the resultant cellular materials, the latter may therefore present a bimodal micro/macroporous or meso/macroporous structure with controlled macropore size distribution. Materials with such hierarchical porosity are very much sought after, due to their combination of high surface area and interconnectivity, thereby leading to improved adsorption and diffusion of molecules. Thus, bimodal carbon foams are expected to be good gas adsorbents, catalyst supports, or filters.

Furthermore, the electrical conductivity of these materials naturally affords interesting electromagnetic (EM) properties. The latter are expected to be tuned by the macroporous structure, especially when the cell sizes are comparable to the wavelength of the EM waves interacting with them. Carbon foams or any other three-dimensional (3D) periodic carbon architectures are therefore of particular interest in the GHz range, and may behave as photonic crystals in microwaves [25,26,27]. In this work, EM properties were therefore investigated with an emphasis on the effect of cell size.

## 2. Materials and Methods

### 2.1. Materials

#### 2.1.1. Preparation of Spherical Templates

Paraffin spheres were prepared according to a procedure described elsewhere [28]. Briefly, molten paraffin (melting point > 60 °C) was suspended in a 5 wt % aqueous solution of polyvinyl alcohol (PVA) at 70 °C. The paraffin/PVA solution blend was vigorously stirred over 10 min, and then icy water was poured into the suspension for solidifying the paraffin spheres. The latter were subsequently washed with distilled water for removing the residual PVA, and then left to dry for 24 h. Finally, the spheres were separated by sifting into seven classes of size ranges: 100–200 µm, 200–300 µm, 300–400 µm, 400–500 µm, 500–600 µm, 600–800 µm, and 800–1000 µm, using a standard series of sieves having mesh sizes ranging from 100 µm to 1000 µm. The corresponding average diameters were thus assumed to be 150 µm, 250 µm, 350 µm, 450 µm, 550 µm, 700 µm, and 900 µm, and the final carbon materials based on RF resin using such templates were therefore called CRF150, CRF250, CRF350, CRF450, CRF550, CRF700, and CRF900 respectively.

#### 2.1.2. Preparation of RF Cellular Monoliths and Derived Carbon Monoliths

Resorcinol (R) was first dissolved in water (W) at an R/W molar ratio of 0.086, and then a 37% aqueous solution of formaldehyde (F) was added so that the R/F molar ratio was 0.5. Gelation of the obtained mixture was catalysed by addition of sodium carbonate (C) in such an amount that the R/C molar ratio was 100:1. The RF solution was then mixed for 10 min to obtain a homogeneous solution. In the meantime, paraffin spheres with desired ranges of sizes were installed in close-compact packing inside glass vials. They were next heated at 40 °C for 20 min, and then cooled down to room temperature. Doing this, the spheres stuck to each other at their contact points.

The aforementioned RF solution was then used to fill glass vials containing close packings of paraffin spheres that were consolidated as explained before, and which were kept under vacuum over 2 h to ensure the complete evacuation of air. After this time, the vials were hermetically closed and placed in an oven at 50 °C for 72 h to ensure complete gelation and ageing of the RF resin. Since the latter had a solid content of 35 wt %, strong and rapidly cross-linked hydrogels were obtained.

Next, the as-obtained RF—paraffin composite materials were removed from their vials and placed in Erlenmeyer flasks, which were filled with petroleum ether for dissolving paraffin. The petroleum ether was replaced by pure one on a daily basis, and after one week, the resultant porous organic monoliths were dried at 85 °C in air for three days, and finally carbonised at 5 °C min^−1^ up to 900 °C (2 h dwell time) under pure nitrogen flowing at 100 mL min^−1^. As a result, the present materials can be considered as cellular carbon xerogels.

### 2.2. Characterisation

#### 2.2.1. Thermogravimetric and Elemental Analyses

Thermogravimetric analysis (TGA) was performed with a STA 449 F3 Jupiter^®^ thermobalance (Netzsch, Germany) using 50 mg of monolithic RF sample placed in an alumina crucible. The sample was heated at 10 °C min^−1^ up to 900 °C under pure argon flowing at 50 mL min^−1^. Prior to TGA measurements, the furnace chamber with the sample inside was outgassed and flushed three times with pure argon to remove any residual air.

The elemental composition of carbon monoliths was determined by use of a Vario EL Cube (Elementar, Germany) in terms of wt % of C, H, O, N, and S. Since all of the samples were prepared from the same resin, only one representative sample was investigated.

#### 2.2.2. Density and Porous Structure

The bulk density, *ρ_b_* (g cm^−3^), of the porous CRF carbon monoliths was calculated as the mass of the samples divided by their volume; the latter was measured from their geometrical dimensions using a digital calliper. Helium pycnometry was used to measure the skeletal density, *ρ_s_* (g cm^−3^). Prior to measurements, samples were crushed in a mortar and evacuated in vacuum at 80 °C for 24 h, so that errors due to possible closed porosity could be avoided. The porosity, *Φ* (dimensionless), and the specific total pore volume, *V_p_* (cm^3^ g^−1^), were then estimated from bulk and skeletal densities, according to the following equations:

(1) $$\Phi =1-\frac{\rho_{b}}{\rho_{s}},$$

(2) $$V_{p}=\frac{1}{\rho_{b}}-\frac{1}{\rho_{s}}$$

Additionally, similar samples were tested in monolithic form (i.e., not ground as before) by helium pycnometry for estimating the percentage of open cells according to:

(3) $$open\;cell\;\%=\frac{Skeletal\;density\;of\;the\;RF\;carbon\;foam}{Skeletal\;density\;of\;finely\;ground\;RF\;carbon\;foam}\times 100$$

The microporous and mesoporous structure of the CRF materials was investigated by nitrogen and carbon dioxide adsorption measurements at −196 °C and 0 °C, respectively, using an ASAP 2020 automatic adsorption apparatus (Micromeritics, Norcross, GA, USA). The samples were outgassed for 48 h under vacuum at 250 °C before any adsorption experiment. The Brunauer–Emmett–Teller (BET) area, *A_BET_* (m^2^ g^−1^), was determined from the BET equation [29] that was applied to the adsorption branch of the isotherms. In order to choose the range of relative pressure *P*/*P*_0_ for fitting the BET equation to the experimental adsorption data, the quantity *V_N_*_2_ × (1 − *P*/*P*_0_), where *V_N_*_2_ is the adsorbed nitrogen volume at a given value of *P*/*P*_0_, was plotted as a function of *P*/*P*_0_ and starting at *P*/*P*_0_ = 0.01. The maximum value of *P*/*P*_0_ for fitting the BET equation was the one such that *V_N_*_2_ × (1 − *P*/*P*_0_) reached its maximum. The micropore volume, *V_DR,N_*_2_ (cm^3^ g^−1^), was determined by application of the Dubinin–Radushkevich equation [30] to the nitrogen isotherms. The micropore volume, which was based on CO_2_ adsorption, *V_DR,CO_*_2_ (cm^3^ g^−1^), was also obtained by application of the Dubinin–Radushkevich equation [30] to CO_2_ adsorption isotherms, giving information on micropores that were hardly accessible to nitrogen molecules due to diffusional resistances at −196 °C. The total pore volume, *V*_0.97_ (cm^3^ g^−1^), which was measurable by nitrogen adsorption, was defined as the volume of liquid nitrogen corresponding to the amount adsorbed at a relative pressure *P*/*P*_0_ = 0.97 [21].

Pore size distributions (PSDs) were determined by application of the non-local density functional theory (NLDFT) combining both nitrogen and carbon dioxide adsorption data. The PSDs were calculated with the SAIEUS^®^ software provided by Micromeritics [31]. The PSDs were then used to calculate the surface area, *S_NLDFT_* (m^2^ g^−1^), the ultramicropore volume, *V_uµ,NLDF_*_T_ (cm^3^ g^−1^) (corresponding to pore diameters <0.7 nm), the micropore volume, *V_µ,NLDF_*_T_ (cm^3^ g^−1^) (corresponding to pore diameters <2 nm), and the total pore volume, *V_NLDF_*_T_ (cm^3^ g^−1^).

The macroporosity and the broadest part of the mesoporosity, as well as the corresponding macro/mesopore size distribution, were assessed by mercury porosimetry using an AutoPore IV 9500 device (Micromeritics). First, the sample holder with the material inside was evacuated and filled with mercury in a low-pressure chamber within the pressure range 0.001–0.24 MPa. Then, the sample holder was filled with the sample, and mercury was transferred to a high-pressure chamber, and the pressure inside was increased from 0.24 MPa to 414 MPa. The volume of mercury that was forced to penetrate in the sample holder was recorded as a function of time, and the pore diameters *D* (nm) were calculated by application of Washburn’s equation:

(4) $$D=\frac{-4\gamma cos\theta }{P},$$

where *γ* (485 mJ m^−2^) is the surface tension of mercury, *θ* (140°) is the contact angle between mercury and the material, and *P* (MPa) is the intrusion pressure. Equation (4) shows that pores as narrow as 3.6 nm can be probed at the highest available pressure of 414 MPa.

#### 2.2.3. Optical and Electron Microscopy

**Figure 1:**
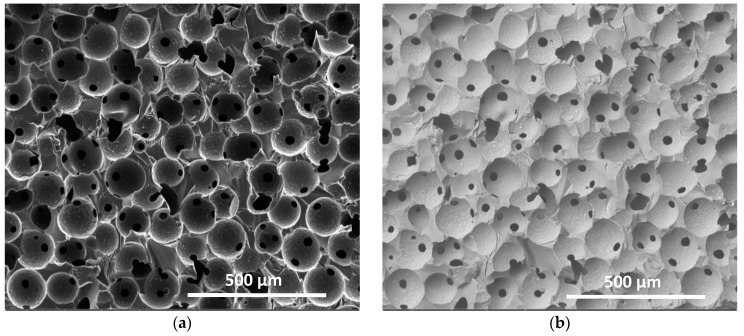
Scanning electron microscopy (SEM) pictures of the CFR250 sample: (**a**) with secondary electrons (SE) detector; (**b**) with backscattered electrons (BSE) detector. Images of type (**a**) and (**b**) were used for measuring cell sizes and window sizes, respectively.

Paraffin spheres were observed with a LV100ND Eclipse Nikon microscope (Tokyo, Japan) equipped with a 5MPix CCD camera to control the shape and the diameter of the spheres. Concerning cellular carbon monoliths, cell structure and average size, as well as average window diameter, i.e., the size of the circular holes connecting the cells, were determined by scanning electron microscopy (SEM) observations with a FEI Quanta 600 microscope (Hillsborough, OR, USA). For that purpose, CRF samples were placed on a carbon-coated sample holder and sputtering-coated under vacuum with an extremely thin carbon layer in a metallisation system. For all of the samples, two modes of observations using detectors of secondary electrons (SE) and backscattered electrons (BSE) were used. SE indeed highlight the topological contrast, and were thus the most suitable for estimating the average cell diameters, whereas BSE were the best for visualising cell windows and measuring their diameters. Figure 1 shows one example, justifying the use of two different electron detectors. Average cell and window diameters were estimated with Image Pro-Plus 6.0 software (Rockville, MD, USA), based on a collection of more than 200 cells and windows. After calibration, the software automatically provided the average sizes of selected pores.

#### 2.2.4. Electrical and Electromagnetic Measurements

**Figure 2:**
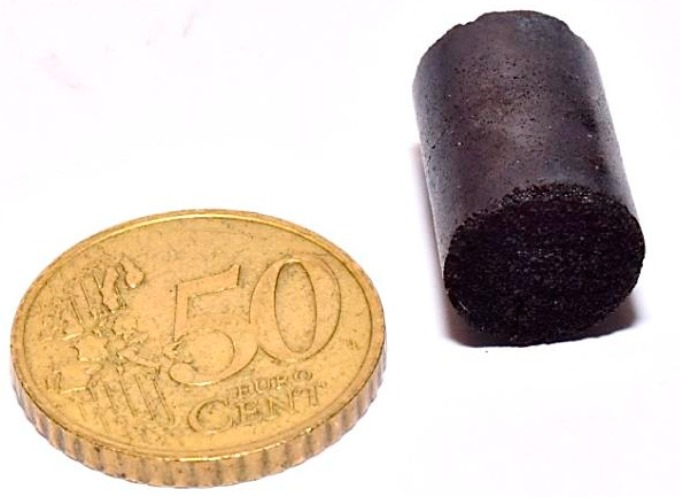
Typical cellular carbon monolith sample (here CRF450) obtained after pyrolysis and used as such for electrical conductivity and bulk density measurements.

The direct current (dc) electrical conductivities of cellular carbon monoliths were measured using a standard two-point technique [32]. Cylindrical pieces of carbon monoliths such as those shown in Figure 2, i.e., the same as those used for determining the bulk density, were placed between two parallel flat silver contacts orthogonal to the cylinder axis. The resistances of the wires and the contact effects were found to be negligibly small with respect to those of the investigated samples, so that the electrical resistance of the whole system, *r* (Ω), was assumed to be that of the materials. The conductivity *σ* (S m^−1^) of the latter was thus calculated from Equation (5):

(5) $$\sigma =\frac{4h}{\pi rd^{2}},$$

where *d* (m) and *h* (m) are the diameter and the height of the cylindrical samples, respectively.

Microwave measurements were carried out with a scalar network analyser R2-408R (ELMIKA, Vilnius, Lithuania), including a sweep generator, waveguide reflectometer, and indicator unit (personal computer). Such a device allowed the reconstruction of the constituent electromagnetic parameters (real and imaginary parts of permittivity: *ε’* and *ε”*, respectively) in the 26–37 GHz frequency range (Ka-band). For that purpose, the waveguide cross-section was 7.2 mm × 3.4 mm. The frequency stability of the oscillator was controlled by a frequency meter, and was as high as 10^−6^. The power stabilisation was provided at the level of 7.0 mW ± 10 μW, and the measurement range of EM attenuation was from 0 to −40 dB. Basic measurement errors of EM attenuation over the range 0 to −25 dB and −25 to −40 dB were *δθ* = ±(0.6 + 0.06*θ*) and *δθ* = ±(−0.4 + 0.1*θ*), respectively, where *θ* is the measured EM attenuation and *δθ* is the margin of error. Rod-like samples with a diameter of *d* = 0.4 mm or higher have been studied. The samples were installed perpendicularly into the waveguide. The electromagnetic (EM) responses of the composites were measured as ratios of transmitted/input (*S*_21_) and reflected/input (*S*_11_) signals. The effective permittivity was recalculated from the *S*-parameters using methods that have been thoroughly detailed elsewhere [33,34].

For EM shielding measurements, 2-mm thick parallelepiped samples were precisely cut to fit the cross-section of the waveguide. Reflectance (*R*), transmittance (*T*), and absorbance (*A*) were calculated from *S*-parameters according to Equations (6)–(8), respectively:

*R* = *S*_11_^2^, (6)

*T* = *S*_21_^2^*,* (7)

*A* = 1 − *R* − *T* (8)

## 3. Results and Discussion

### 3.1. Carbonisation Behaviour and Resultant Composition

**Figure 3:**
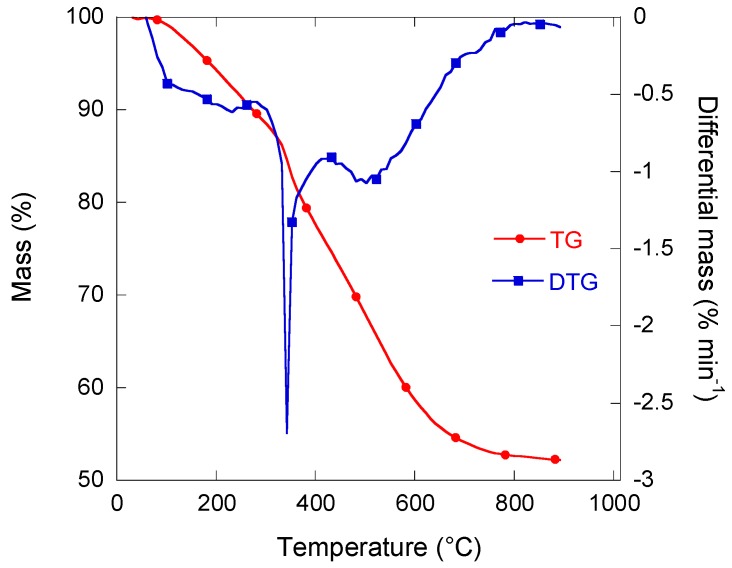
Mass thermogravimetry (TG) and differential mass thermogravimetry (DTG) curves of organic RF350 porous monolith.

Thermogravimetry (TG) and differential thermogravimetry (DTG) curves of a representative sample of RF monolith (RF350) are presented in Figure 3. The mass loss was found to be equal to 47%, and three main contributions could be observed: from 50 °C to 250 °C, from 250 °C to 400 °C, and from 400 °C to 700 °C. A broad DTG band was seen between around 80 °C and 220 °C, corresponding to the release of moisture and light organic compounds. Two DTG peaks also appeared at 350 °C and 500 °C, indicating a maximum rate of mass loss at these temperatures. The main peak in the range 200–400 °C was attributed to the breakage of C–O bonds, whereas some C–H bonds broke at temperatures higher than 500 °C [22].

The results of the elemental analysis of the sample CRF350 are gathered in Table 1. Since all of the formulations were the same for the other materials, which only differed by the size of paraffin spheres, no additional analyses were carried out. The carbon content was quite high, about 95%, with a non-negligible amount of oxygen, almost 4%, which is expected in glasslike carbon due to the phenolic nature of the precursor.

### 3.2. Porosity and Pore Texture Parameters

#### 3.2.1. Overall Porosity

Helium pycnometry led to values of skeletal density ranging from 1.98 cm^−3^ to 2.04 g cm^−3^, without any trend from CRF150 to CRF900. These small differences are typical, and are within the uncertainty of this kind of measurements. Taking an average of 2.00 g cm^−3^, and using the values of bulk density listed in Table 2, overall porosity, open porosity, and total specific pore volume were calculated by the application of Equations (1), (3) and (2), respectively. These quantities are given in Table 2.

Increasing the diameters of paraffin beads used as templates produced a gradual increase of bulk density, from 0.38 cm^−3^ to 0.59 g cm^−3^. This finding may be explained by the thicker and thicker pore walls that were produced by bigger and bigger sacrificial spheres touching each other at contacts, whose dimensions then increased much less than the diameter of the templates. Smaller spheres indeed presented proportionally higher contact areas with their neighbours than bigger ones in the corresponding close packing, and therefore could come relatively closer to each other. Wax spheres of higher diameters only led to comparatively much smaller contact areas, and there was consequently a much broader gap to be filled in by the resin that was poured between them. As a result, the density increased with the diameter of the paraffin spheres, so that the corresponding overall porosity decreased and presented moderately high values: 74–83%. The porosity of most of the carbon monoliths is indeed most of the time typically 10–20% higher [1,3,14]. The porosity of the CRF materials, however, was essentially open, around 90%, as seen in Table 2.

#### 3.2.2. Microporous Structure

**Figure 4:**
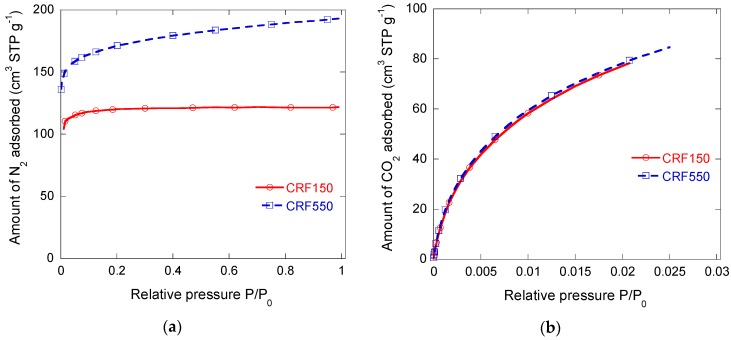
Adsorption isotherms measured for CRF150 and CRF550 samples: (**a**) N_2_ at −196 °C; (**b**) CO_2_ at 0 °C.

Nitrogen adsorption experiments were difficult, requiring extremely long equilibrium times. This was most likely due to the presence of extremely narrow microporosity and nitrogen diffusion limitations at −196 °C. Two representative isotherms are shown in Figure 4a. They were indeed type Ia for CRF150 and Ib for CRF450, and according to the International Union of Pure and Applied Chemistry (IUPAC) classification, i.e., are typical of microporous materials. High BET surface areas, as far as carbon xerogels are concerned, were observed, within the range 480–660 m^2^ g^−1^ as seen in Table 3, which is far higher than in most carbon foams for which the area is only the geometrical, external one, and is indeed in the order of magnitude of 1 m^2^ g^−1^ [3,14,35]. However, the unusually long equilibrium times made these results questionable.

Carbon dioxide isotherms were thus built for the same samples, and both were almost identical to each other; see Figure 4b. This finding strongly supports the lack of accessibility of N_2_ molecules to very narrow micropores. It also suggests that the microporosity created during pyrolysis was logically not influenced by the size of the paraffin beads. Higher values of *V_DR,CO_*_2_ than those of *V_DR,N_*_2_ were determined, confirming important nitrogen diffusion limitations. In the presence of such small micropores, CO_2_ adsorption therefore appears to be an absolutely necessary characterisation tool that is complementary to nitrogen adsorption. Carbon dioxide indeed assesses pores narrower than 1.2 nm, while N_2_ does not allow a correct characterisation of pores narrower than 0.7 nm. For sample CRF150, *V_DR,N_*_2_ was slightly higher than *V*_0.97_, which is due to the overestimation of the micropore volume by application of the Dubinin–Radushkevich (DR) equation. Herein, the DR model was exclusively used to compare *V_DR,CO_*_2_ and *V_DR,N_*_2_, and confirms that there are very narrow pores because *V_DR,CO_*_2_ > *V_DR,N_*_2_. The most reliable determination of the micropore volume and the total pore volume is given by application of the NLDFT model.

**Figure 5:**
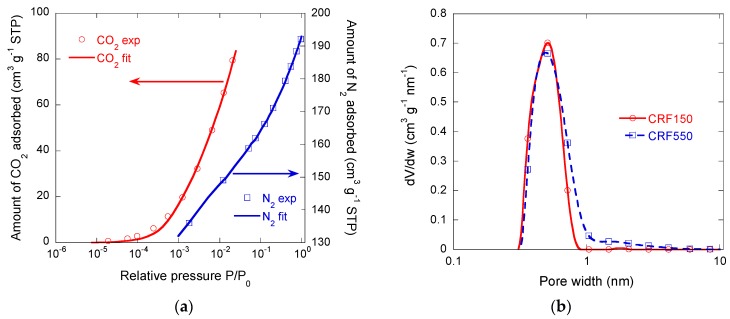
(**a**) Fits by SAIEUS^®^ software (full lines) of the experimental N_2_ (blue squares) and CO_2_ (red circles) isotherms of the CRF550 sample; (**b**) corresponding pore size distribution; and comparison with that of CRF150 sample.

Figure 5a shows an example of fitting of N_2_ and CO_2_ isotherms with SAIEUS^®^ software for the case of sample CRF550. Fitting both isotherms simultaneously allowed obtaining the pore size distribution (PSD) and, from such PSD, the various pore volumes that are given in Table 3. *V_µ,NLDFT_* was lower than *V_DR,CO_*_2_ due to the overestimation of the microporosity by application of the DR equation. However, *V_NLDFT_* was higher than *V*_0.97_ because the latter only determined the volume of pores filled by nitrogen, whereas NLDFT took both N_2_ and CO_2_ isotherms into account. The corresponding data are given in Table 3. Figure 5b shows the NLDFT PSDs obtained for samples CRF150 and CRF550. Both are very similar, and confirm that most of the microporosity was only accessible to CO_2_ molecules. The main part of the microporosity is indeed present in the range of pore size from 0.4 nm to 1 nm, with a main peak centred on 0.5 nm. Narrow mesopores, with a maximum at a width of 2.7 nm, were also observed in Figure 5b, although their corresponding volume was much lower.

These data thus show that CRF150 and CRF550 samples are both fundamentally ultramicroporous, although CRF 550 showed a slightly higher surface area due to wider pores, as suggested by N_2_ adsorption, and accounting for the slight slope of its isotherm at *P*/*P*_0_ higher than 0.1 (see again Figure 4a). However, such small differences did not merit examining the full range of samples since, as explained before, only the cell size was expected to change; the phenolic resin precursor of the carbon and the gelation and pyrolysis conditions was always the same.

The pore texture characteristics presented in Table 3, as well as the PSDs, suggest that these materials might be used as carbon molecular sieves [36]. This is especially the case for CRF150, which combines the highest total pore volume (see again Table 2) for an efficient transport throughout the material, the highest ultramicroporous fraction, and a negligible mesoporosity. Moreover, the corresponding pore volumes are typical of those of the carbon molecular sieves that are presently available on the market, for instance for chromatographic applications.

#### 3.2.3. Macroporous Structure

**Figure 6:**
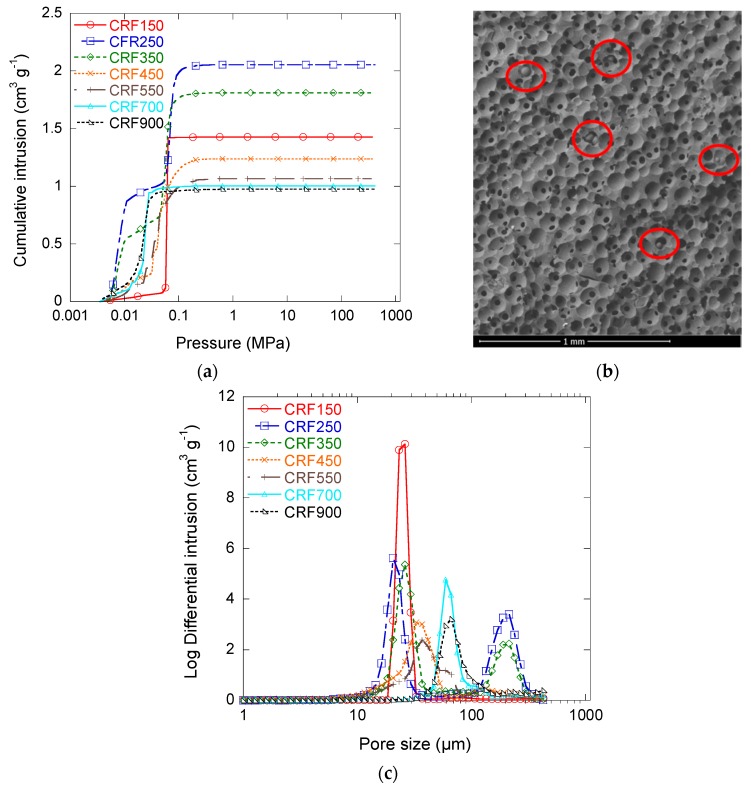
(**a**) Mercury intrusion curves of all cellular carbon monoliths; (**b**) SEM pictures with BSE detector of CRF150 sample: cells filled with carbon spheres are highlighted by red circles; (**c**) pore size distributions derived from (**a**).

The mercury intrusion curves of the CRF materials are given in Figure 6a. Two regions separated by a quasi-plateau could be observed for the series of samples from CRF250 to CRF550. The onset of the first intrusion, occurring at a pressure of about 0.05 MPa, corresponds to the filling of the largest macropores, i.e., of cells produced by paraffin beads and/or cracks and surface heterogeneities. As mercury only probes the entrance diameters of pores, it is expected that the volume intruded in the first step corresponded to voids that are directly accessible, i.e., not only throughout smaller connections. The other samples—CRF150, CRF700, and CRF900—presented only one single frank intrusion step. In this case, all of the cells were probably accessible through small entrances with well-defined diameters. Since the porosity decreased when the paraffin beads’ size increased, the total intruded volume decreased from CRF250 to CRF900, as expected.

Sample CRF150 was the exception to this trend, as it presented a much lower intruded volume than expected. This finding can be explained when considering the SEM images that were obtained with the backscattering electron detector, as shown in Figure 6b. Some cells are filled with small spheres that could effectively partly prevent the intrusion of mercury. Such spheres were probably made by the polymerisation of RF resin at the surface of individual paraffin beads, and are therefore expected to be hollow after pyrolysis. Their presence therefore did not much influence the bulk density of CRF150 sample, which remains the lightest of the series, as already observed in Table 2.

**Figure 7:**
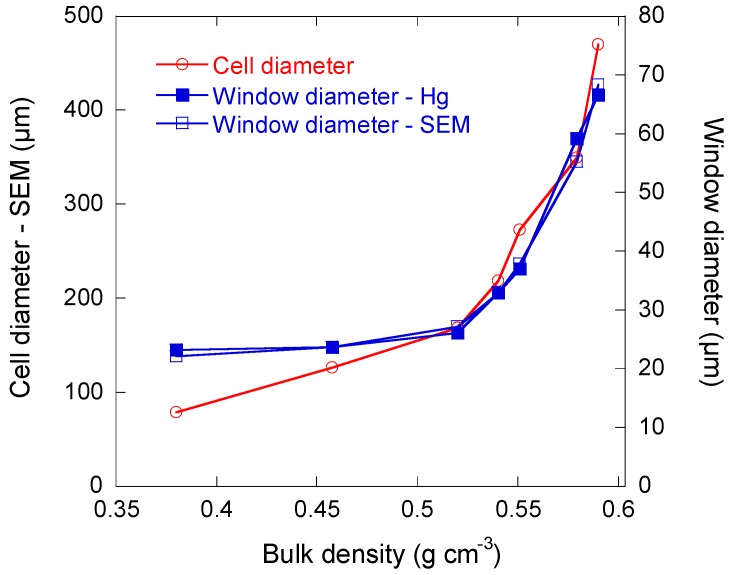
Average cell diameters derived from SEM observations, and window diameters measured either by mercury porosimetry or by SEM studies.

The corresponding pore size distributions were calculated by the application of Equation (4). The data reported in Figure 6c are not ∂*V*/∂*w* versus *w*, where *V* and *w* stand for the intruded Hg volume and for the pore width, respectively, but ∂*V*/∂log*w* versus *w*, which is known as “log differential intrusion”. This kind of presentation is known to emphasise the pores of higher diameters, which otherwise would be hardly visible [37]. As explained above, cells were only accessible through windows in CRF150, CRF700 and CRF900 samples, with peak diameters at 23 µm, 58 µm, and 62 µm, respectively. Both CRF250 and CRF350 samples presented a peak at 200 µm, attributable to directly accessible cells. Samples CRF250, CRF350, CRF450, and CRF550 also presented peak window sizes of 23 µm, 27 µm, 33 µm, and 38 µm, respectively. Therefore, increasing the size of the paraffin beads in the initial formulation made the window diameters increase. Such behaviour is logical when considering that bigger paraffin spheres led to higher contact area and hence to higher window sizes between spherical cells. The trends are shown in Figure 7.

The changes of window size as a function of bulk density are thus totally opposite to what is usually found in traditional foams, i.e., in materials that have been expanded by the release of gas within the liquid formulation before hardening [35,38]. Indeed, in usual foams, lower porosity is associated with smaller pores, whether the latter are windows or cells, and power laws have been found to describe quite correctly this kind of behaviour. However, it should be recalled that the present materials were obtained by hard-templating, and therefore, whereas the structure looks like that of foams, the way it changes with porosity is totally different. The same applies to cell size, which was measured from SEM observations, and which should decrease according to a power law of the density with a negative exponent [35,38,39]. Again, Figure 7 evidenced a totally opposite behaviour, which was directly related to the special preparation mode of those materials.

**Figure 8:**
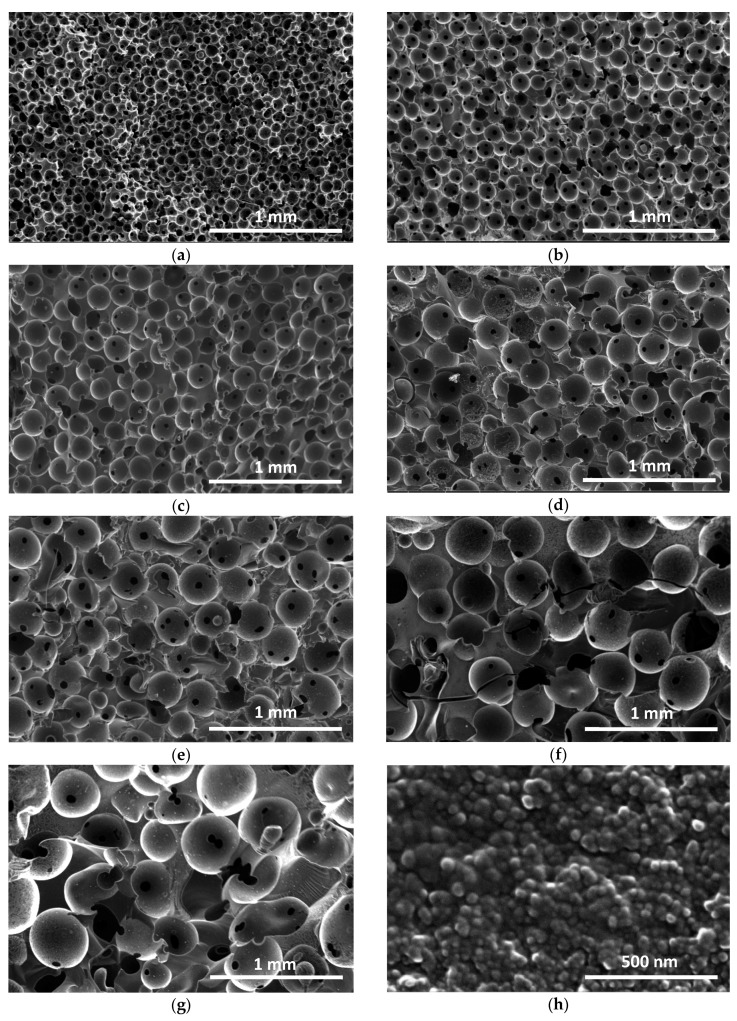
SEM pictures of cellular carbon monoliths prepared with paraffin beads of different sizes: (**a**) CRF150; (**b**) CRF250; (**c**) CRF350; (**d**) CRF450; (**e**) CRF550; (**f**) CRF700; (**g**) CRF900. (**h**) View of a cell wall of CRF350 sample at high magnification.

**Figure 9:**
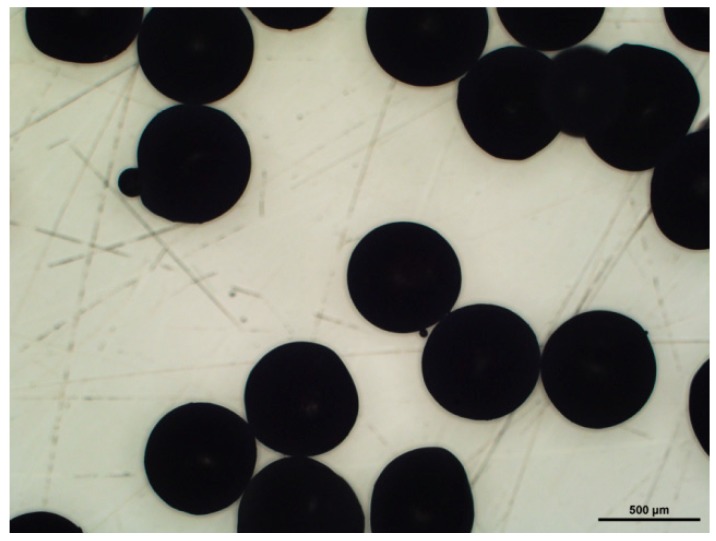
Optical micrograph of paraffin beads having an average particle diameter of 450 µm, and obtained between sieves of mesh sizes 400–500 µm. The scale bar represents 500 µm.

The SEM images of the full series of CRF samples are given in Figure 8. The carbon structure of all of the materials properly reproduced the replica of close packings based on paraffin spheres having increasing sizes. Although small initial differences in the diameter of these spheres prevented a perfect ordering of the packings, all of the carbon monoliths exhibited spherical cells with quite well-calibrated sizes that were connected with their nearest neighbours through at least one circular window in each direction of the space. The samples that were prepared with the biggest templates presented less uniform structures because of the difficulty of maintaining very even morphologies for such big paraffin spheres. The latter indeed presented smaller particles glued to them such as those seen in Figure 9, and were impossible to remove by the simple sieving procedure used here.

Figure 8 clearly shows that the present monoliths were such that bigger cells were systematically associated with thicker cell walls and higher densities. As emphasised above, this finding is completely opposed to that which is found in materials obtained by conventional foaming, for which bigger cells always correspond to lower densities [40]. However, the present behaviour was already observed in other cellular carbon monoliths prepared, not by hard-templating, but rather by soft-templating and explained accordingly [41]: small templates pack with small gaps, while big ones produce broad gaps and hence thick cell walls. A very good agreement between the window sizes estimated from mercury porosimetry and SEM can be observed in Figure 7, as the two sets of values were nearly identical. Cell walls that were observed at higher magnification revealed the typical nodular structure of carbon gels derived from phenolic resins [42,43,44,45,46,47], as shown in Figure 8h. The average nodule diameter was close to 40 nm, and such a structure explains the significant amount of micropores, which are known to be located inside the nodules, whereas mesopores rather correspond to internodular spaces [48].

A last remark can be made about the controllability of the cellular macrostructure. In standard carbon foams, the density and pore structure are controlled through the amount of blowing agent; higher amounts lead to lower densities and bigger cells (unless special care is taken for de-correlating density and cell size, as did Letellier et al. [39]). However, homogeneity and final structural properties may also depend on ambient conditions and/or on the way of preparation. In the case of the present materials, the porous structure can be fully controlled by using templates of a suitable size, especially if an efficient sieving system can be used, thanks to which a very narrow and well-defined pore size distribution can be obtained. As far as carbon materials are concerned, such control is less easy because of the shrinkage naturally occurring during the final pyrolysis step.

**Figure 10:**
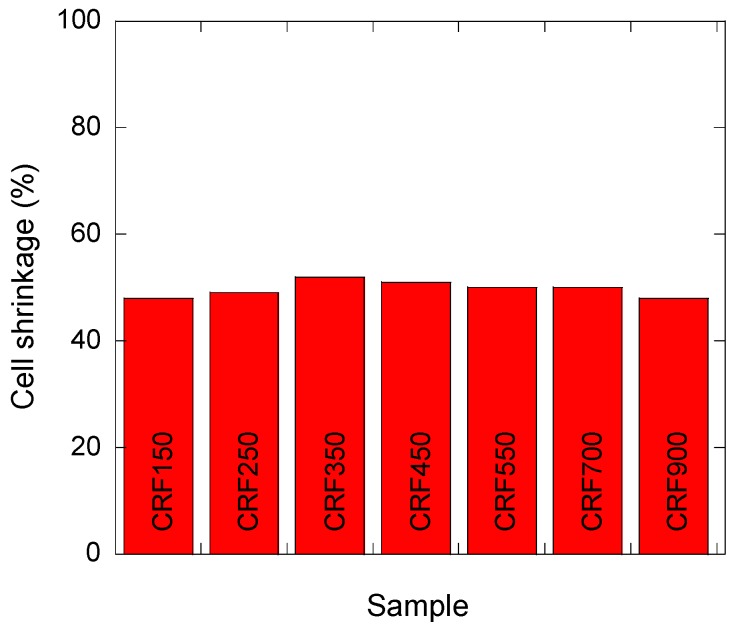
Cell shrinkage calculated for all of the materials, thanks to which carbon monoliths of predictable cell size can be prepared.

However, the present CRF materials did not present such drawbacks, as all of the measured cell shrinkages were found to be nearly the same, as shown in Figure 10. Therefore, knowing the value of shrinkage after pyrolysis, it becomes easy to choose proper diameters for the paraffin beads in order to obtain the desired cell size in the final carbon monoliths. The small differences observed from one sample to another in Figure 10 are likely due to the average (median) sizes of the beads and corresponding cells that were used for these calculations, whereas the reality dealt more with distributions than with unique values of diameters.

### 3.3. Electrical Properties

**Figure 11:**
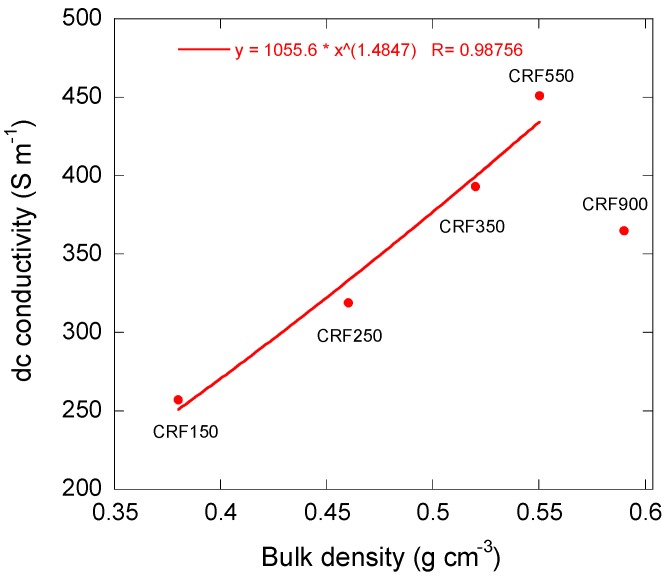
Electrical conductivity of cellular carbon monoliths. The curve corresponds to the fit of Equation (9) to the first four data points.

The obtained values of dc conductivity are shown in Figure 11. It can be seen that the conductivity increased monotonously until reaching a density that corresponded to sample CRF550, above which it decreased. The former behaviour is expected, since the porosity indeed decreased from CRF150 to CRF550, and has been already reported in carbon foams derived from phenolic resins [25,49]. The dc electrical conductivity thus only depends on density, not on cell size. Moreover, the simplest law describing the electrical behaviour of metallic foams [50] was successfully fitted to the experimental data in this range of densities:

*σ* ~ *ρ_b_^α^*, (9)

where *σ* is the dc conductivity, *ρ_b_* is again the bulk density, and *α* is an exponent whose value is expected to be 3/2. The fit led to a value of 1.48, as shown in Figure 11, which is in excellent agreement with the prediction of Ashby et al. [51]. At higher density, the law was no more obeyed, which was probably due to the inhomogeneity of the samples already suspected from Figure 8f,g.

### 3.4. Electromagnetic Properties

**Figure 12:**
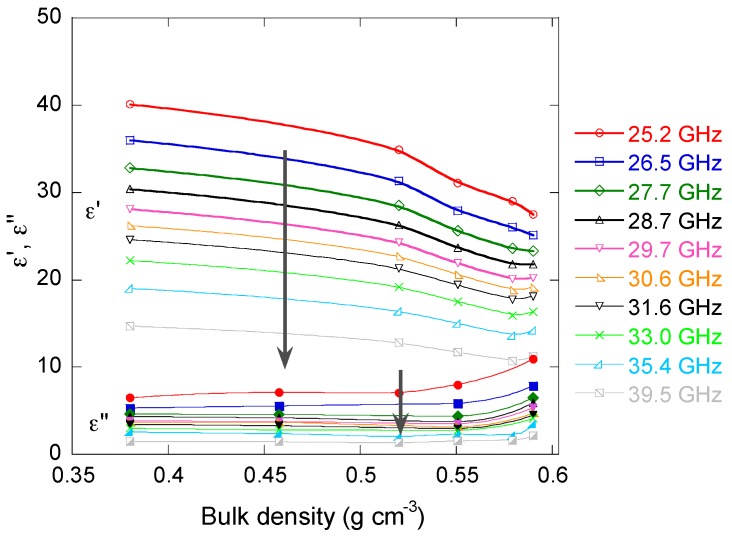
Frequency dependence of real (*ε’*) and imaginary (*ε”*) parts of the effective permittivity as a function of bulk density. The arrows indicate the increase of frequency at which the measurements were carried out.

The changes of effective permittivity of the samples in the microwave frequency range are presented in Figure 12. The real part *ε’* decreased when the bulk density increased, whereas the imaginary part *ε”* parts increased. Such behaviour may be understood as follows. The real part of dielectric permittivity is proportional to the capacitance *C* of the material. For instance, for a plane capacitor, it reads *C* = *ε’ ε*_0_ *S*/*d*, where *S* is the cross-section area of the sample, and *d* is the thickness. Thus, the capacitance of the material decreases when its density increases. Now, the porous carbon monoliths may be considered as resistor–capacitor (RC) circuits, and in this case, the capacitance may be roughly associated with cells filled with air. An increase of density thus corresponds to a decrease of the volume fraction of air, and therefore to the observed decrease of *ε’*. The increase of the imaginary part *ε”* is explained by that of electrical conductivity with density, since *σ* = *ε*_0_ *ε” ω*, where *ω* is the angular frequency.

The same trend was observed as a function of cell size (not shown). The latter finding is obvious, since cell size increased with bulk density, as seen in Figure 8, and since it was shown recently [52] that *ε’* and *ε”* don’t depend on the cell size of carbon foams having constant bulk density in the Ka-band. However, both *ε’* and *ε”* monotonically decreased when the frequency increased, and their absolute value was quite high, with *ε’* as high as 40.1 + *i*6.47 for CRF100 sample at 25.2 GHz. Therefore, high absolute values of permittivity in combination with other parameters such as low density, thermal conductivity, and mechanical strength make these monoliths potential candidate materials for shielding applications.

**Figure 13:**
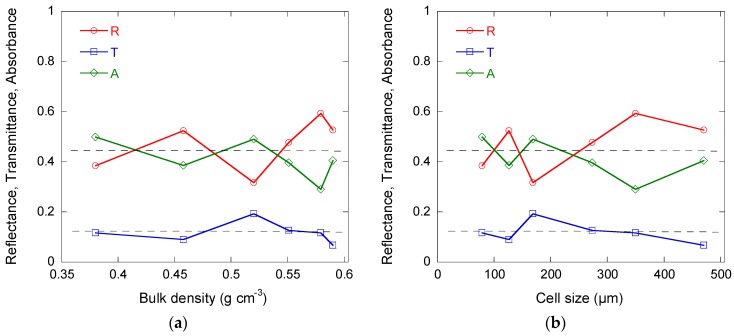
Electromagnetic (EM) properties of cellular carbon monoliths as a function of (**a**) bulk density, and (**b**) cell size: reflectance (*R*), absorbance (*A*), and transmittance (*T*) at 30 GHz. The dashed lines are just guides for the eye and show the main trends. The measurement error is at the level of 3–5%.

The dependence of reflectance (*R*), absorbance (*A*), and transmittance (*T*) on bulk density and on cell size at 30 GHz is presented in Figure 13a,b, respectively. All of the samples demonstrated high shielding efficiency due to their combination of high reflectance and high absorbance, which were both close to 45%. As a result, the average transmittance was low and close to 15%. No special dependence on cell size or density was observed, and in the limit of the scattering of the results, *R*, *A,* and *T* can be considered constant. An equivalent behaviour was observed in the same range of frequencies for carbon foams having a bulk density higher than 0.2 g cm^−3^ and cell sizes close to 150 µm [52].

The independence of the reflectance on cell size can be related to the cells being connected with each other through very narrow circular windows. Due to such a structural feature, EM waves thus poorly penetrate the materials, since the skin depth of the glassy carbon skeleton is at the level of 200 µm [53]. The absorption of EM waves is related to Ohmic losses, and consequently to the conductivity. Now, the latter was found to be almost independent of density and cell size, and the absorbance is nearly constant. Finally, since both reflectance and absorbance demonstrate negligible dependence, then the same applies to transmittance.

Finally, the following can be added. In contrast with true carbon foams, which are mostly reflective in the microwave range, and reticulated ones, which can be highly absorptive [52] at certain cell sizes corresponding to geometrical resonances versus wavelengths [53], the present cellular carbon monoliths have a low level of transmittance while only 2-mm thick. However, this level is caused by both high absorption and high reflection levels (>40%). In cases where high absorption ability is required, e.g., for electromagnetic compatibility (EMC) applications, the simplest way is to use the so-called Salisbury screen effect (1/4 of wavelength), which minimises reflectance and leads to almost perfect absorption. 

## 4. Conclusions

New, fully controllable, cellular glassy carbon monoliths were prepared and described. Controllability is of major importance for designing materials that have more predictable and repeatable properties in some relevant applications, giving them higher performances and efficiency. Besides, materials having a bimodal, macro/microporous structure, provide an advantage with respect to conventional carbon foams, as both high surface area and excellent transport throughout the porosity are required in specific applications that are related to filtration, gas separation, catalysis, or energy storage. The present cellular carbon monoliths, which are based on resorcinol-formaldehyde resin as a precursor and paraffin beads of controlled size as templates, had porosities within the range of 75–85%, and presented both narrow cell size distributions and narrow microporosity with very high surface areas compared to most macrocellular solids. It was shown that, knowing the initial diameter of the paraffin spheres as well as the typical shrinkage after carbonisation, it is possible to obtain samples with desired cell sizes.

It should be emphasised here that due to the special way by which these materials were prepared, i.e., using sacrificial spherical templates, the cell size increased with density. This is a very special feature; it is totally opposite to what is found in solid foams, where bigger cells correspond to lower density. It was therefore particularly relevant to investigate their electrical and electromagnetic properties. The latter were measured in static regime and in the Ka-band, respectively.

The electrical conductivity followed a power law of density, and the corresponding exponent was very close to 3/2, as predicted by the simplest model of closed-cell foams. This relationship supported the former observation that electrical conductivity only depends on porosity, and not on cell size. However, the observed dependence was no more obeyed as soon as the homogeneity of the samples was not high enough, especially at the highest density and cell size. In the Ka-band, the real and imaginary parts of the permittivity decreased and increased with density, respectively. This feature was explained by the corresponding lower fraction of porosity. The high permittivity observed, combined to lightness, suggest that these materials might be used for EM shielding. Transmission of EM waves was indeed low, close to 15%, and was nearly constant in the full range of investigated densities.

Since this type of cellular carbon monoliths is much easier to control and is stiffer than other cellular of reticulated carbon foams with mostly open porosity, the most practical EM application is electromagnetic compatibility. One may easily produce a ¼ wavelength structure made of this type of porous monoliths, reaching almost zero reflection and almost perfect absorption with an extremely lightweight (0.4–0.6 g cm^−3^) and relatively thin (around 2.5 mm for 30 GHz) carbon layer, being at the same time resistant to heat and mechanically robust, due to the glassy carbon from which it is made.

## Figures and Tables

**Table 1:** Elemental analysis of one representative cellular carbon monolith sample: CRF350.

Sample	C (wt %)	H (wt %)	O (wt %)	N (wt %)	S (wt %)
CRF350	94.87	0.55	3.81	0.96	0.03

**Table 2:** Density and porosity of cellular carbon monoliths.

Sample	Bulk Density (g cm^−3^)	Overall Porosity (%)	Open Porosity (%)	Total Specific Pore Volume (cm^3^ g^−1^)
CRF150	0.38	83.1	89.8	2.19
CRF250	0.46	79.7	89.3	1.74
CRF350	0.52	76.9	89.3	1.48
CRF450	0.54	76.0	90.7	1.41
CRF550	0.55	75.5	88.4	1.37
CRF700	0.58	74.3	88.9	1.28
CRF900	0.59	73.8	88.0	1.25

**Table 3:** Pore texture characteristics of cellular carbon monoliths derived from Figure 5.

Sample		CRF150	CRF550
N_2_ adsorption	*A_BET_* (m^2^ g^−1^)	482	663
	*V_DR,N_*_2_ (cm^3^ g^−1^)	0.20	0.25
	*V*_0.97_ (cm^3^ g^−1^)	0.19	0.30
CO_2_ adsorption	*V_DR,CO_*_2_ (cm^3^ g^−1^)	0.29	0.29
	*S_NLDFT_* (m^2^ g^−1^)	844	976
	*V_uµ,NLDFT_* (cm^3^ g^−1^)	0.199 (93%)	0.199 (64%)
	*V_µ,NLDFT_* (cm^3^ g^−1^)	0.215 (100%)	0.274 (88%)
	*V_NLDFT_* (cm^3^ g^−1^)	0.215	0.312
